# Sexual dysfunction after cancer: gender differences, tumor-specific patterns, and implications for sexual medicine practice

**DOI:** 10.3389/or.2026.1807376

**Published:** 2026-04-13

**Authors:** Isabella Bisceglia, Fortunato Morabito, Guglielmo Ferrari, Filippo Giovanardi, Carmine Pinto, Amelia Ceci, Antonino Neri, Lucia Mangone

**Affiliations:** 1 Epidemiology Unit, Azienda USL-IRCCS di Reggio Emilia, Reggio Emilia, Italy; 2 Associazione “Vittorio Lodini per la Ricerca in Chirurgia” di Reggio Emilia, Reggio Emilia, Italy; 3 Medical Oncology Unit, Azienda USL-IRCCS di Reggio Emilia, Reggio Emilia, Italy; 4 Health Promotion Staff and Gender Medicine Group, Azienda USL-IRCCS di Reggio Emilia, Reggio Emilia, Italy; 5 Scientific Directorate, Azienda USL-IRCCS di Reggio Emilia, Reggio Emilia, Italy

**Keywords:** biopsychosocial model, cancer survivorship, gender differences, psychosexual interventions, sexual dysfunction, sexual medicine

## Abstract

**Background:**

Sexual health is a central component of wellbeing, identity, and intimate relationships, yet it remains insufficiently addressed in cancer care. Cancer treatments disrupt sexual functioning through interacting physical, hormonal, psychological, and relational mechanisms, leading to persistent and often under-recognized sexual dysfunction among survivors.

**Aim:**

To synthesize current evidence on cancer-related sexual dysfunction, assessment strategies, and therapeutic interventions, with a focus on gender differences, tumor-site specificity, and implications for sexual medicine practice.

**Methods:**

This narrative review integrates evidence from population-based studies, clinical guidelines, and systematic reviews addressing sexual dysfunction across cancer types. Gender-specific patterns and biopsychosocial mechanisms were examined to inform assessment and management within sexual medicine and survivorship care.

**Results:**

Women commonly experience multidimensional and frequently “invisible” sexual difficulties, including reduced desire and arousal, orgasmic dysfunction, dyspareunia, vaginal atrophy, body image disturbance, and fertility-related distress. Men more often present with overt functional impairments, particularly erectile and ejaculatory dysfunction following prostate and other male cancer treatments. Existing assessment tools capture selected aspects of sexual function but often fail to reflect the full biopsychosocial complexity of post-cancer sexuality. Effective management requires integrated interventions combining medical and pharmacological therapies, physical rehabilitation, psychosexual and couples counseling, and structured communication models. Tailored, gender- and tumor-specific approaches embedded within multidisciplinary survivorship pathways are essential, including culturally competent care for sexual and gender minority patients.

**Conclusion:**

Sexual dysfunction is a prevalent and clinically relevant consequence of cancer. Comprehensive assessment and personalized, multidisciplinary interventions are essential components of high-quality sexual medicine care for cancer survivors.

## Introduction

A cancer diagnosis—and the subsequent sequence of surgery, chemotherapy, radiotherapy, endocrine therapy, and follow-up—constitutes more than a physical burden (1). It disrupts personal identity, relationships, bodily integrity, and sexuality. Core quality of life (QoL) dimensions, including sexual health, fertility, and body image, often receive limited clinical attention despite their relevance for patients and partners (2, 3). In younger women with breast cancer, fertility loss, breast/chest alterations, and treatment-related sexual dysfunction are strongly associated with impaired QoL and relational wellbeing (4). These findings underscore the need for pre-treatment counselling that explicitly addresses sexual and reproductive concerns and actively involves partners in decision-making (2, 5, 6). A multidisciplinary approach integrating pharmacological, psychological, and rehabilitative interventions is consistently recommended to address sexual and couple concerns comprehensively (7).

Comparable challenges occur in gynecologic cancers, where treatment-induced menopause and anatomical or hormonal changes profoundly affect sexual function and female identity, necessitating early and sustained clinician–patient communication (8). In male cancers, consequences for sexual function, self-perception, and sexual activity are substantial yet insufficiently investigated (9).

Sexual consequences extend beyond reproductive cancers. Patients with head and neck tumours frequently report impaired intimacy and sexuality, often exacerbated by post-treatment aesthetic or functional changes (10). Sexual dysfunction arises from interacting physical, endocrine, and psychological/relational mechanisms that vary according to tumour site, treatment modality, gender, and individual circumstances. Physical factors include surgical alteration of breast, pelvic, or genital structures, producing scarring, sensory loss, and positional discomfort. Pelvic or chest-wall radiotherapy may induce mucosal injury, vascular damage, fibrosis, or stenosis, contributing to dyspareunia or compromised erectile function (11). Chemotherapy-related fatigue, neuropathy, and deconditioning further limit sexual functioning.

Endocrine mechanisms are pivotal: desire, arousal, lubrication, erection, and orgasm depend on intact hormonal signalling. Ovarian suppression or failure, bilateral oophorectomy, chemotherapy-induced menopause, anti-estrogen and androgen deprivation therapies disrupt these pathways, leading to vaginal dryness, atrophy, dyspareunia, and arousal or orgasmic dysfunction (12). Although often invisible, these changes substantially affect sexual wellbeing.

Psychological and relational dimensions compound these effects. Altered body image, diminished sexual self-concept, fertility loss, depression, anxiety, and fear of recurrence may suppress intimacy, while shifts in partner roles—particularly transitions toward caregiver—can strain relationships.

Across cancer populations, body-image disturbance and emotional distress are strong predictors of sexual dysfunction (13). Many symptoms remain concealed, contributing to persistent under-recognition. Despite growing evidence, major communication barriers persist. Limited training in sexual health, clinician discomfort, and cultural taboos continue to hinder systematic integration of sexuality into oncology and palliative care (7). Structured communication models, including PLI SS IT (Permission, Limited Information, Specific Suggestions, Intensive Therapy), alongside targeted educational interventions, have shown promise in improving clinician confidence and patient-reported outcomes (7). We conducted a non-systematic literature search using PubMed and Scopus (period 2000-2025) with the terms ‘cancer survivorship’, ‘sexual dysfunction’, and ‘sexual health’ to enhance transparency in source selection.

This narrative review is intended to support sexual medicine clinicians involved in the care of cancer survivors and synthesizes evidence on sexual health disturbances in cancer patients, highlighting differences by tumour site and gender. It describes underlying mechanisms, distinguishes visible from invisible sequelae, and reviews current assessment and management strategies, emphasizing the need for a multidisciplinary, patient-centred approach to improve recognition and care across oncology and survivorship settings.

## Methods: narrative review approach

This manuscript presents a narrative review of the literature on sexual dysfunction in cancer survivors, with particular attention to tumor-site specificity, gender differences, underlying biopsychosocial mechanisms, and clinical implications for sexual medicine practice. Although not a systematic review, source selection was guided by clinical relevance and methodological quality. A non-systematic literature search was conducted using PubMed/MEDLINE and Scopus to identify relevant publications from January 2000 to March 2025. The search strategy combined Medical Subject Headings and free-text terms related to cancer survivorship and sexual health, including “cancer survivorship,” “sexual dysfunction,” “sexual health,” “sexual wellbeing,” “genitourinary syndrome of menopause,” “erectile dysfunction,” “dyspareunia,” “body image,” and “psychosexual interventions.” Reference lists of selected articles were also screened manually to identify additional pertinent studies. Publications were considered eligible if they addressed sexual function or sexual wellbeing in adult patients with cancer or cancer survivors. Priority was given to population-based studies, clinical guidelines, systematic reviews, meta-analyses, and randomized or observational clinical studies with clear relevance to sexual health outcomes. Studies focused exclusively on pediatric populations, non-cancer conditions, or without specific relevance to sexuality were excluded. Given the narrative nature of the review, no formal quality scoring system was applied; however, studies were selected based on methodological robustness, clinical relevance, and contribution to sexual medicine practice. Relevant data were extracted qualitatively and synthesized thematically rather than quantitatively. The analysis focused on cancer type, treatment modality, mechanisms of sexual dysfunction, gender-specific patterns, assessment instruments, and therapeutic strategies. Findings were interpreted within a biopsychosocial framework, distinguishing between visible anatomical changes and less apparent or “invisible” sexual sequelae, and emphasizing their implications for clinical assessment, multidisciplinary management, communication strategies, and inclusive care for sexual and gender minority patients.

## Sexuality across cancer types: the importance of tumor‐site specificity

One of the most neglected yet clinically vital issues is how sexual problems differ depending on the anatomical and biological site of the tumor. Tumor-site specificity is central to sexual medicine, as mechanisms of dysfunction, symptom visibility, and therapeutic targets vary substantially The distinctions are not minor: they influence mechanisms of dysfunction, patient experience, partner dynamics, and clinical intervention. Moreover, an often‐overlooked element is the visibility or invisibility of sexual sequelae—changes that are obvious to the patient/partner or to the clinician, and changes that are hidden but potent. Here we explore three major groups: breast cancer, ovarian/fallopian tube cancer, and uterine/endometrial (and other gynecologic) cancers.

## Breast cancer

Breast cancer represents a paradigm in which sexuality, body image, fertility, and treatment side effects converge. On the one hand, there are visible changes such as mastectomy, lumpectomy, reconstruction or the absence thereof, surgical scars, breast asymmetry, altered chest sensation, lymphedema, and hair loss from chemotherapy. These visible alterations can powerfully influence self-perceived femininity, attractiveness, and sexual agency, often contributing to psychological distress and impaired intimate relationships (14–16). On the other hand, invisible changes encompass chemotherapy-induced ovarian failure or premature menopause, endocrine therapies reducing estrogen and androgen levels, vaginal dryness, dyspareunia, decreased lubrication and arousal, and alterations in orgasmic capacity (16–18). These invisible sequelae can substantially reduce sexual desire and satisfaction, impacting quality of life even when external appearance is preserved. The interplay of visible and invisible changes underscores the importance of a multidimensional approach to sexual health in breast cancer survivors, integrating medical, psychological, and relational interventions (14, 15). From the anatomical/physical perspective, surgical removal of the breast or part thereof can disrupt the sense of sexual bodily integrity and pleasure. The breast is a part of feminine sexual identity for many, and its alteration or removal may evoke loss of attractiveness, changed partner perception, reluctance to engage in intimate touch, and avoidance of sexual activity (19). The presence of lymphedema or limitations in shoulder mobility after axillary dissection further constrains sexual positions and comfort. Hormonal and physiologic changes are significant but less visible. Many women treated for breast cancer undergo premature menopause or ovarian suppression, leading to abrupt loss of estrogen, decreased vaginal lubrication, increased pain during intercourse, diminished arousal, and sometimes reduced orgasmic function (20). A systematic review of genitourinary syndrome of menopause in breast cancer survivors highlighted substantial impairment in sexual satisfaction due to vaginal dryness, atrophy, and dyspareunia (21). These internal changes, while not outwardly apparent, may have a profound impact on sexual function and relationship intimacy. Psychologically and relationally, body image disruption plays a central role. For many women, the altered chest becomes a signifier of illness, of changed femininity, of lost normalcy. Sexual self‐esteem can decline, desire can wane, and partner communication may falter. Younger women face the added burden of fertility concerns, which intertwine with sexual identity (“am I still a woman?”, “can I be a mother?”) and relational future planning. Mangiardi-Veltin et al. (6) emphasise that sexual rehabilitation in breast cancer must engage body image, endocrine side‐effects, fertility, and partner dynamics. In a large ageing cohort in the United Kingdom, Jackson et al. (22) found that while sexual activity among cancer survivors did not differ significantly from controls, women with cancer reported significantly greater dissatisfaction (18.2% vs. 11.8%; P = 0.034) and were more likely to report arousal and orgasm difficulties if diagnosed <5 years (55.4% vs. 31.8%; 60.6% vs. 28.3%). This illustrates that despite preserved sexual activity, the quality of sexual experience can be substantially impaired. In practice, the combination of visible and invisible changes means that breast cancer survivors may assume that because the breast “looks” reconstructed or cosmetically managed, sexual issues are resolved—but the internal hormonal and genitourinary consequences may persist, unspoken and untreated. Clinical practice must therefore integrate both visible/anatomical functions and invisible/hormonal dynamics.

## Ovarian and fallopian tube cancer

In the context of ovarian and fallopian tube cancers, the sexual sequelae often differ qualitatively from those in breast cancer. Because the primary tumour is internal, many of the effects are less externally visible but deeply consequential in hormonal, anatomical, and relational domains. Physically, surgical treatment often involves bilateral salpingo-oophorectomy, hysterectomy, omentectomy, and lymphadenectomy. The removal of ovaries results in abrupt loss of ovarian hormone production, including estrogen, progesterone, and intra-ovarian androgens, which can substantially affect sexual desire, arousal, and lubrication (23). Pelvic surgery may lead to adhesions, scarring, pain, and discomfort during intercourse, as well as changes in pelvic space that affect sexual positions and comfort, even though externally the genital region may appear unchanged (23, 24). These internal and less apparent sequelae highlight the need for a multidisciplinary approach that integrates hormonal, anatomical, and psychosocial factors to optimize sexual health and quality of life in women treated for ovarian cancer. Hormonal and physiological disturbances are particularly prominent in patients undergoing therapy for gynecologic malignancies. The abrupt onset of menopause—especially when surgically or chemotherapy-induced in premenopausal women—leads to vaginal atrophy, reduced lubrication, dyspareunia, diminished sexual desire, and often a decline in the quality or frequency of orgasm.

A recent study of 53 female cancer survivors who had undergone fertility preservation reported sexual dysfunction in 60.4% of participants, with diminished ovarian reserve (AMH <1.1 ng/mL) present in 52.6% of women under 40 years; domains most affected included lubrication and orgasm, measured using the Female Sexual Function Index (FSFI) (25). Similarly, clinical practice guidelines note that among gynecologic cancer survivors, up to 90% experience bothersome sexual dysfunction, primarily related to internal genital and hormonal sequelae such as vaginal dryness, dyspareunia, and reduced sexual desire (26).

Recent evidence highlights the high prevalence and treatability of genitourinary syndrome of menopause (GSM) in breast and gynecologic cancer survivors. A retrospective study of 185 women comparing vaginal estriol, vaginal DHEA, and ospemifene showed significant improvements in sexual function across all measures without endometrial changes or cancer recurrence, supporting short-term safety. Ospemifene offered slightly superior dyspareunia relief. Overall, 92.4% of patients reported symptomatic improvement, confirming GSM as both common and highly responsive to targeted therapies (27).

These internal, largely non-visible changes have significant consequences for sexual function, intimacy, and overall quality of life in survivors of gynecologic cancers. Population-level data confirm the substantial burden of hormonal and genitourinary dysfunction: a systematic review and meta-analysis of female Arab cancer survivors reported high rates of sexual dysfunction, with vaginal dryness in 19.8%–54.2% and dyspareunia in 22.2%–65% of women (28). Although not specific to ovarian cancer, these patterns parallel those seen in gynecologic malignancies, where abrupt menopause, endocrine deprivation, and pelvic surgery drive significant genitourinary morbidity. The review also noted persistent gaps in communication and unmet informational needs, often exacerbated by cultural stigma, highlighting that many sexual sequelae remain both anatomically and socially invisible.

When examined alongside clinical data, a clear pattern emerges: genitourinary and sexual dysfunction are highly prevalent, effective treatments exist, yet they remain underused. Sexual dysfunction in gynecologic malignancies is common, driven by internal hormonal and anatomical changes that are not externally visible, and often goes unaddressed despite evidence-based management options. The invisibility of sexual changes in ovarian cancer underscores the need for proactive counselling, pre-treatment discussions of contraception/fertility and hormone loss, attention to vaginal health, and post-treatment referral to sexual rehabilitation, pelvic floor physiotherapy, atrophy management, and couples therapy. Significant research gaps remain, especially intervention trials focused specifically on this subgroup.

## Uterine (endometrial) and other gynaecologic cancers

Uterine (endometrial) cancer is increasingly diagnosed worldwide, and the sexual implications parallel those in ovarian/fallopian tube cancers—but with their own unique pattern. Standard treatment often comprises hysterectomy (removal of the uterus) with or without bilateral salpingo-oophorectomy, followed in many cases by adjuvant pelvic radiotherapy and/or brachytherapy. The removal of the uterus and sometimes the ovaries, along with pelvic radiation, leads to sexual sequelae that are both visible (surgical scars, change in abdominal contour, perhaps absence of uterus visible via imaging only) and invisible (vaginal shortening, vault alterations, fibrosis, dryness, hormonal loss, reduced orgasmic sensation) (29). Physically, vaginal vault changes—particularly after radiotherapy—can lead to stenosis, reduced elasticity, and pain during intercourse. Although the vulva and external genitalia may remain unchanged, the internal architecture is significantly altered. Because these changes are hidden, patients may struggle to articulate discomfort, and clinicians may fail to probe. A 2024 literature review (30) noted that women with gynecologic cancers consistently reported poorer sexual function, pain, anxiety about intimacy, and body image concerns—even in the absence of visible external changes. Hormonal consequences are substantial when ovaries are removed or irradiated; early menopause, loss of androgenic support, decreased lubrication, and diminished sexual desire/pleasure follow. The sense of lost fertility and altered sexual role as a *woman* can be especially poignant in younger patients. Yet because the outer appearance may appear unchanged, the sexual impact is often unrecognized. Relationally and psychologically, women with uterine cancer may feel that “nothing appears different” but “I no longer feel the same.” Body image may alter even when external anatomy is unaffected—for instance, feelings of “lost womb,” “lost possibility of children,” and changed sexual self-image. Partner reactions may be muted because outward signs are minimal, thereby depriving the patient of visible acknowledgement of her change and complicating intimacy. From a clinical perspective, the challenge is that whereas in breast cancer the “visible” changes are obvious and prompt conversations, in uterine/gynaecologic cancers, the invisible changes require deliberate enquiry. Interventions must include vaginal dilator programmes, moisturisers/lubricants, pelvic floor therapy, hormone replacement when safe, body image counselling, and couple intimacy work. Research remains limited: few long‐term cohorts have followed sexual recovery, and few RCTs have targeted these survivors with tailored interventions.

**FIGURE 1:**
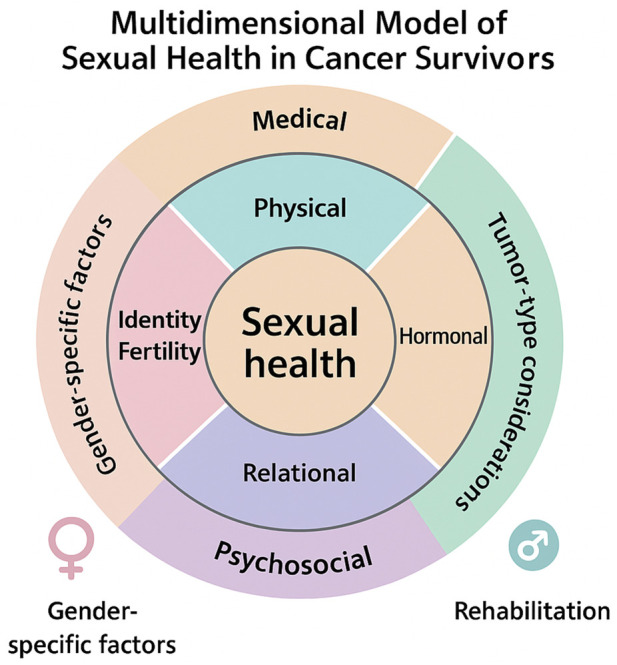
Visible and Invisible Sexual Sequelae Across Cancer Types. This infographic compares *visible* and *invisible* sexual health changes associated with breast, ovarian/fallopian tube, and uterine/endometrial cancers. Visible impacts (e.g., mastectomy, surgical scars, hysterectomy) are contrasted with less visible but clinically significant sexual sequelae such as treatment-induced menopause, vaginal dryness, decreased lubrication, dyspareunia, and altered arousal or libido. The figure highlights how tumour site, treatment modality, and anatomical disruption shape distinct patterns of sexual dysfunction, underscoring the need for tailored assessment and management in survivorship care.

Vulvar and vaginal tumors are rare gynecological malignancies but have significant clinical and psychosocial consequences. Beyond oncological outcomes, growing evidence indicates that these tumors and their treatments negatively affect sexual function and quality of life in affected women. Clinical practice and literature suggest that women treated for vulvar cancer often experience sexual dysfunction. In a prospective study with 24-month follow-up, only 9.8% of patients were sexually active at the time of diagnosis, a percentage that increased to 22.8% after 2 years; 73.5% of women reported dissatisfaction with their sex life and many expressed a need for clinical support regarding sexuality (31). Surgical intervention, particularly inguinal lymphadenectomy and extended vulvectomy, is associated with deterioration of sexual function. Forner et al. reported that inguinal lymphadenectomy is related to reduced postoperative sexual function (32). Long-term studies confirm that survivors exhibit lower sexual activity than the general population, often experiencing persistent genital pain (33). Beyond physical results, perception of body image and psychological wellbeing play a fundamental role in sexual satisfaction. Women with vulvar cancer often report concerns about body image and feelings of inadequacy, contributing to reduced sexual life and impaired interpersonal relationships (34, 35). Pelvic radiation therapy, commonly used in vaginal cancers, can cause vaginal stricture, dryness, and fibrosis, which increase the risk of dyspareunia and sexual difficulties. Although studies specific to vaginal cancer are limited due to its rarity, evidence for cervical and vulvar cancers suggests similar sexual complications following pelvic treatments. Evidence indicates that vulvar and vaginal cancers, particularly when treated surgically or with radiation therapy, negatively affect sexual function, quality of life, and body image. It is essential that oncology treatment pathways integrate the assessment of sexual function and dedicated support interventions to improve psychosocial outcomes in women affected by these tumors. Figure 1 illustrates how sexual health consequences differ across major female cancer types, distinguishing visible anatomical changes from the less apparent but highly impactful sexual symptoms that often remain under-recognized in clinical practice.

## Prostate and other male cancers

Prostate cancer survivors frequently experience significant sexual dysfunction as a consequence of both the malignancy and its therapies; indeed, post-treatment sexual sequelae such as erectile dysfunction, loss of libido, and ejaculatory changes (e.g., anejaculation) are among the most prevalent survivorship concerns (36). Radical prostatectomy often causes immediate erectile dysfunction due to cavernous nerve injury, along with loss of ejaculation, whereas radiotherapy leads to a more gradual decline in erectile function; by approximately 3 years post-treatment, erectile dysfunction rates are comparable between surgery and radiotherapy when no hormonal therapy is added (37). Androgen deprivation therapy (ADT) has the most profound impact, frequently inducing loss of sexual interest and erectile capacity; ADT is associated with a markedly elevated risk of both reduced libido and erectile dysfunction. Younger survivors of testicular cancer likewise face unique challenges: orchiectomy and multimodal therapies can impair fertility, alter body image and self-esteem, and may lead to hypogonadism requiring testosterone replacement (38). These impacts underscore the need for proactive sexual rehabilitation, including pharmacotherapy (e.g., phosphodiesterase type 5 inhibitors), mechanical interventions such as vacuum erection devices or penile prostheses for refractory cases, and psychosexual counseling to support adaptation and improve long-term quality of life.

## Assessment of sexual health in oncology

Despite long-standing recommendations from expert bodies, systematic assessment of sexual health in oncology remains sporadic. The recent narrative review and position statement from the Italian Association of Medical Oncology (AIOM) highlights how sexual dysfunction is under-recognized in practice because screening is infrequent and conversations about sexuality are often avoided or deferred (13).

Routine enquiry should be considered an essential component of quality-of-life care. Several validated tools—such as the FSFI and its cancer-specific adaptations, the IIEF, and measures of body image and sexual adjustment—can support assessment. However, comparative systematic reviews show that commonly used instruments often overemphasize desire and satisfaction while insufficiently capturing arousal, orgasm, body image, and relational factors, leaving key aspects of post-treatment sexuality under-assessed (39, 40). Although cancer-adapted FSFI versions address some gaps, no single measure fully reflects the biopsychosocial complexity experienced by survivors (41).

Barriers to routine assessment arise at both clinician and patient levels. Clinicians frequently face limited training, time constraints, disease-focused priorities, and discomfort discussing sexual issues, while patients may experience shame, cultural stigma, or fear of judgment. A national Italian survey further demonstrated significant deficits in provider awareness and preparedness to address sexual health in women and sexual-gender-minority patients, underscoring the need for education and structured clinical pathways (42).

Assessment should be individualized according to tumor site and treatment. In breast cancer, evaluation must address body-image changes after mastectomy or reconstruction, altered chest sensation, lymphedema and mobility limitations, endocrine-related genitourinary symptoms, fertility concerns, and partner communication. In ovarian and fallopian tube cancers, abrupt treatment-induced menopause, adhesions, and altered pelvic anatomy necessitate focused enquiry on lubrication, pain, positional difficulties, and loss of fertility. In uterine and other pelvic malignancies, radiation-associated vaginal shortening, fibrosis, and stenosis require targeted assessment of penetration pain, bleeding, and sexual avoidance (43). A comprehensive approach extends beyond penetrative intercourse to include non-penetrative intimacy, masturbation, sexual identity and orientation, partner dynamics, and the emotional significance of sexuality. Combining brief screening questions with selective use of validated tools and subsequent open dialogue improves detection of otherwise hidden and undertreated problems (44).

## Interventions and therapeutic strategies

Managing sexual dysfunction in cancer survivors requires an individualized, tumour-site sensitive, gender-aware and multidisciplinary approach that blends medical treatments, rehabilitation and psychosexual care. Interventions must balance symptomatic benefit with oncologic safety and be embedded within structured communication and survivorship pathways.

## Medical/pharmacological interventions

For women with genital symptoms, non-hormonal therapies (e.g., vaginal moisturisers and lubricants) are often the first-line option, because they carry minimal systemic risk. Vaginal moisturizers and water-based lubricants help maintain tissue hydration and reduce friction, and they are strongly recommended as first-line options in breast cancer survivors who may be cautious about hormonal treatments (45). When these are insufficient, local vaginal oestrogen may be considered. Meta-analyses suggest that vaginal oestrogen in breast-cancer survivors is not associated with an increased recurrence risk: one pooled meta-analysis of observational studies (n = 24,060) reported an odds ratio (OR) of 0.48 (95% CI 0.23–0.98) for breast-cancer recurrence in users vs. non-users of vaginal oestrogen (46). A further meta-analysis of seven studies including 118,659 survivors found no significant increase in recurrence (relative risk [RR] 0.87, 95% CI 0.67–1.11) and observed a reduction in all-cause mortality (RR 0.80, 95% CI 0.75–0.86) among vaginal oestrogen users (47). In cohort data from a Scottish–Welsh registry of 49,237 women with breast cancer, use of vaginal oestrogen after diagnosis was *not* associated with higher breast-cancer-specific mortality (hazard ratio [HR] 0.77, 95% CI 0.63–0.94) (48). Randomised trials of vaginal oestrogen and vaginal DHEA (prasterone) have not shown sustained elevations in systemic oestradiol or increased adverse events over the short term (49).

For men, sexual dysfunction after prostate-cancer surgery or radiation is commonly managed with penile rehabilitation. A meta-analysis of randomized and observational studies found that phosphodiesterase-5 inhibitors (PDE5-is), vacuum erection devices (VEDs), and intracavernosal injections substantially improve erectile function during treatment (odds ratio [OR] = 2.80; 95% CI, 1.93–4.06) and International Index of Erectile Function (IIEF) scores (50). However, the same analysis showed that after washout of PDE5-, there was no clear evidence of spontaneous erectile recovery (OR = 1.03; 95% CI, 0.71–1.48) (50). Early penile rehabilitation using vacuum erection devices (VEDs) has been shown in randomized trials to enhance IIEF scores and reduce penile shrinkage without serious adverse effects (51). Meta-analyses also support the combined use of PDE5i, VED, and intracavernosal injections in increasing erectile function recovery rates, although spontaneous (unassisted) erections remain less common (52). Alternative options for men who do not respond adequately to PDE5i include intracavernosal injections, intraurethral alprostadil, or penile prosthesis; reviews suggest these remain viable treatment paths, especially when initiated early and in a structured rehabilitation program (53). A multidisciplinary approach involving urology and sexual medicine specialists is recommended to tailor treatment plans, set realistic expectations, and optimize long-term outcomes.

## Physical rehabilitation

Pelvic-floor physiotherapy—including muscle training, biofeedback, and dilator therapy—is commonly used to reduce dyspareunia and improve sexual comfort in women after pelvic surgery or radiotherapy. Clinical and observational data support its role in addressing pelvic pain, improving muscle function, and enhancing quality of life. For gynecological cancer survivors, a systematic review found moderate-level evidence that pelvic-floor muscle training (PFMT) plus counseling (sometimes with yoga or core exercises) improves sexual function and health-related quality of life (54). Vaginal dilator therapy is routinely prescribed in oncology protocols to prevent post-radiotherapy stenosis and fibrosis; however, high-quality randomized trial evidence is limited. A Cochrane review concluded that while observational studies suggest an association between dilator use and reduced self-reported vaginal stenosis, the data do not conclusively prove causation (55). In a prospective observational study of 53 gynecologic cancer survivors, use of dilators (started within 3 months after radiotherapy) was linked to reductions in stenosis grade and improvements in sexual quality of life over 12 months (56). A recent interventional physical-therapy program also showed significant increases in vaginal length and diameter, and resolution of stenosis in two-thirds of participants, along with improved pelvic-floor strength and quality of life (57). Qualitative research likewise highlights that structured PFMT, especially with physiotherapist support and biofeedback, is meaningful to survivors and can relieve pelvic pain and enhance sexual health (58).

## Psychosocial and couples therapy

Psychosexual counselling—encompassing cognitive-behavioural therapy (CBT), acceptance/mindfulness-based approaches (e.g., ACT or MBIs), body-image work, and couple therapy—has shown efficacy in improving multiple dimensions of sexual health in cancer survivors. Systematic reviews and meta-analyses of randomized controlled trials show moderate to large effect sizes for psychological interventions on sexual function, satisfaction, relationship quality, and distress (59, 60). Mindfulness-based interventions (MBIs) in particular have produced clinically meaningful improvements in sexual function (e.g., FSFI domains) in cancer survivors, according to a recent meta-analysis (61). Couple-based sex therapy, especially in breast cancer survivors, is effective: it improves sexual functioning, self-image, and relationship satisfaction (62). In prostate cancer survivors, CBT-based interventions have also demonstrated benefits for both sexual function and mental health (63).

## Communication models and care pathways

**FIGURE 2:**
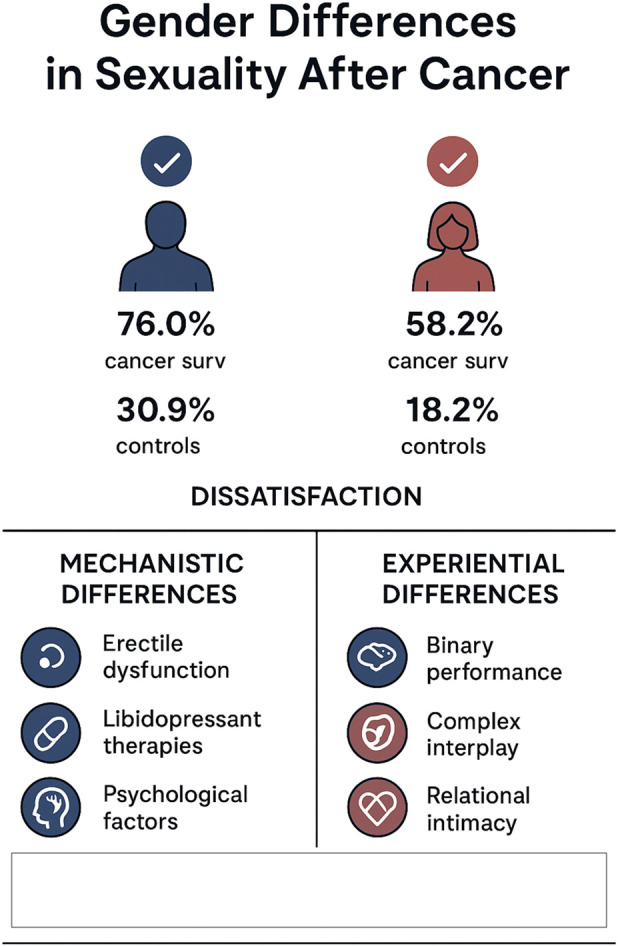
Integrated Sexual Medicine Interventions for Cancer Survivors. This infographic summarizes the principal domains of sexual rehabilitation after cancer treatment, including medical and pharmacological interventions (non-hormonal therapies, local estrogen, PDE5 inhibitors), physical rehabilitation (pelvic floor physiotherapy, biofeedback, dilator therapy), psychosocial and couples-based approaches (CBT, psychotherapy, partner-focused interventions), and structured communication models that integrate sexual health into routine oncology care (PLISSIT model and survivorship care plans). Together, these components outline a multidisciplinary, tumor-site-sensitive, and gender-aware model for managing sexual dysfunction across survivorship.

Structured communication is essential in sexual rehabilitation. The PLISSIT model (Permission, Limited Information, Specific Suggestions, Intensive Therapy) offers a pragmatic framework for clinicians: giving patients permission to raise sexual concerns, providing targeted education, offering tailored suggestions, and escalating to specialist therapy when needed. In oncology settings, use of PLISSIT-based sex counselling has been shown to improve sexual quality of life in breast cancer survivors (64). Embedding sexuality assessment into pre-treatment counselling, active therapy, and survivorship care plans ensures that sexual health is proactively addressed, rather than reactive. Tailored intervention pathways—reflecting tumor type and treatment (e.g., radiation-induced vaginal stenosis vs. genital changes in breast cancer) — should integrate medical, rehabilitative, and psychosexual strategies to provide a holistic, patient-centred approach. A visual overview of the main therapeutic pathways is provided in Figure 2, which outlines the medical, physical, psychosocial, and communication-based strategies that form an integrated approach to managing sexual dysfunction in cancer survivors.

## Gender differences in sexuality after cancer

While sexual health in cancer survivors is often studied separately in men (e.g., prostate/uro-oncology) and women (e.g., breast/gynaecologic cancers), comparative data suggest important gender-specific patterns. In a large, population-based study of older adults (50+ years) from the English Longitudinal Study of Ageing (ELSA), Jackson et al. found that sexual activity levels among cancer survivors were broadly similar to cancer-free controls (men: 76.0% vs. 78.5%; women: 58.2% vs. 55.5%) (22). However, both male and female survivors reported greater dissatisfaction with their sex lives (men: 30.9% vs. 19.8%, P = 0.023; women: 18.2% vs. 11.8%, P = 0.034) (22). Among women diagnosed within the past 5 years, there were significantly higher rates of arousal difficulties (55.4% vs. 31.8%) and orgasm problems (60.6% vs. 28.3%) compared to controls (22). Notably, in that study, men did *not* show a statistically significant change in erectile dysfunction compared to controls (22). These findings suggest that although many cancer survivors maintain sexual activity, the quality of sexual experience—especially for women—may decline, and dissatisfaction can persist. In addition to well-recognized sexual health consequences in cancers directly affecting the genitourinary or reproductive systems, other oncologic populations also experience significant impacts on sexual functioning, desire, intimacy, and overall sexual function. Research shows that patients diagnosed with head and neck cancer often report declines in sexual interest, enjoyment, and intimacy following diagnosis and treatment, with self-reported sexual problems varying widely in prevalence depending on the study population and assessment methods. These changes are frequently mediated not only by physiological sequelae of treatment but also by alterations in body image, psychological distress, and fatigue (65). Similarly, individuals undergoing hematologic cancer therapies, including hematopoietic stem cell transplantation (HSCT) for leukemia, lymphoma, and other blood malignancies, commonly experience reduced libido, diminished frequency of sexual activity, erectile dysfunction, vaginal dryness, and broader sexual dissatisfaction compared to pre-treatment levels. These outcomes have been attributed to a combination of treatment-induced hormonal changes, fatigue, pain, body image concerns, and psychosocial stressors (66). Lastly, the literature highlights a significant scarcity of data addressing sexual health–related problems among LGNT + communities, particularly in the oncological setting. These unmet sexual health needs often compound pre-existing psychosocial, structural, and healthcare-related disparities faced by LGNT + patients, including stigma, minority stress, limited access to culturally competent care, and reduced disclosure of sexual concerns within clinical encounters (67, 68).

## Mechanistic and experiential differences

**FIGURE 3:**
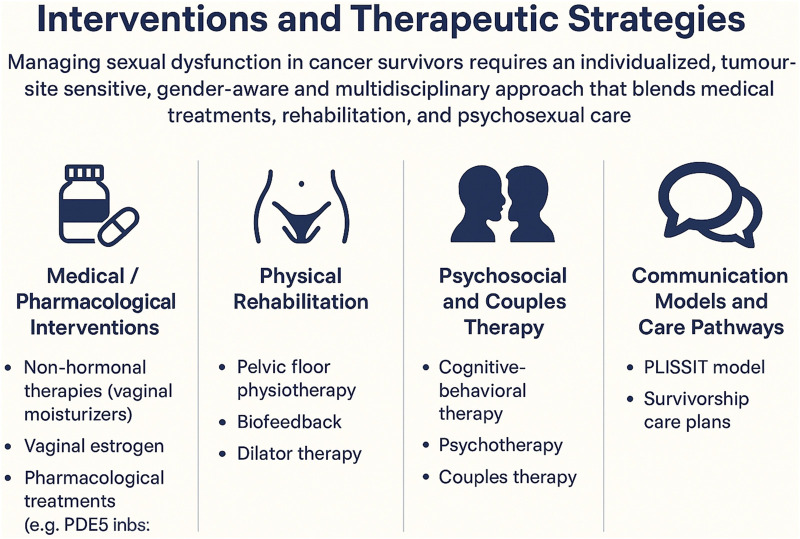
Biopsychosocial Model of Sexual Health in Cancer Survivorship Summary schematic illustrating key gender-specific patterns in sexual activity, dissatisfaction, and mechanisms of sexual dysfunction among cancer survivors. Sexual activity levels in male and female survivors are broadly similar compared with controls, but higher dissatisfaction in both groups, particularly among women. The figure contrasts the more overt, functionally focused sexual changes typically seen in men (e.g., erectile/ejaculatory dysfunction, hormone-therapy–related libido reduction) with the multidimensional, often less visible sexual difficulties reported by women (e.g., arousal, lubrication, orgasm difficulties, pain, and body-image concerns). Psychosocial and relational contributors, more pronounced in women, are also highlighted.

The nature of sexual dysfunction in men often involves more overt, functionally visible deficits: for example, after prostate cancer treatment, neurovascular damage may impair erection; hormone therapies (e.g., androgen deprivation) can also suppress libido; and psychological factors (e.g., concerns about masculinity or performance) frequently contribute (69, 70). In contrast, women’s sexual dysfunction tends to be more multidimensional and less visible: reduced lubrication, atrophy, pain, altered genital sensation, body-image disruption, and fertility concerns can all play roles. Because many of these changes are not externally evident, they may be under-reported by patients and under-recognized by clinicians. From an experiential standpoint, men’s sexual health issues often revolve around binary “performance” metrics (erectile function, ejaculation), whereas women’s sexual distress is more likely to reflect a complex interplay of desire, arousal, lubrication, orgasm, pain, self-image, relational intimacy, and identity (e.g., fertility or motherhood) (71). These gender differences argue for tailored approaches in survivorship care: Men may benefit from early screening for erectile and ejaculatory dysfunction, hormone level monitoring, and psychosocial support around masculinity, role change, and sexual performance. Women require broader screening that encompasses body image, vaginal/genitourinary health (lubrication, pain), fertility counseling, partner intimacy, and the psychosocial impacts of hormonal or identity changes. Communication is especially important. For instance, qualitative and survey data suggest that women are less likely than men to be asked about sexual concerns in oncology settings, leading to under-screening (72, 73). Relationally, the partner’s role matters: in women, partner support and reactions to body-image changes (e.g., after mastectomy) can buffer distress; in men, open dialogue about erectile dysfunction and identity transitions (e.g., from “lover” to “patient”) can be vital. Couples-based interventions may help all genders navigate these shifts. Long-term survivorship studies also underscore these gendered dynamics. For example, a cohort study of cancer survivors 5 and 10 years after diagnosis found that almost half perceived their sexual life as less satisfying than before cancer (74). Physical symptom burden (pain, fatigue), comorbidities, relational satisfaction, and social support were among the strongest correlates of lower sexual satisfaction, emphasizing how sexual health after cancer is deeply intertwined with broader physical and psychosocial wellbeing. Moreover, in specific cancer types, women’s sexual distress is closely linked with vaginal symptoms and relational concerns. For example, in cervical cancer survivors, vaginal symptoms, worries about pain, and body-image issues were significantly associated with sexual distress (75). Overall, sexual dysfunction after cancer manifests differently in men and women, reflecting both biological and psychosocial factors (Figure 3).

Men frequently experience more overt functional deficits, such as erectile or ejaculatory dysfunction, which are relatively straightforward to recognize and often fit neatly into established biomedical treatment pathways. Women, by contrast, tend to face a more complex and multifaceted pattern of sexual changes. These may include reduced desire, arousal difficulties, lubrication and orgasm issues, pain during intercourse, and disruptions in body image or sexual identity. Because many of these changes are less visible and intertwined with psychosocial dimensions, women’s sexual distress is often underreported, underrecognized, and more susceptible to the influence of cultural silence around female sexuality. Despite these differences, both men and women benefit from comprehensive, holistic approaches to sexual health in the oncology setting. The optimal care model requires that intervention priorities, communication strategies, and clinical pathways be thoughtfully tailored to each patient, taking into account gender, tumor type, treatment history, and relational context. Such a personalized approach ensures that the physical, emotional, and relational aspects of sexual health are addressed, acknowledging the nuanced ways cancer can affect sexuality across genders.

## Conclusion

**FIGURE 4:**
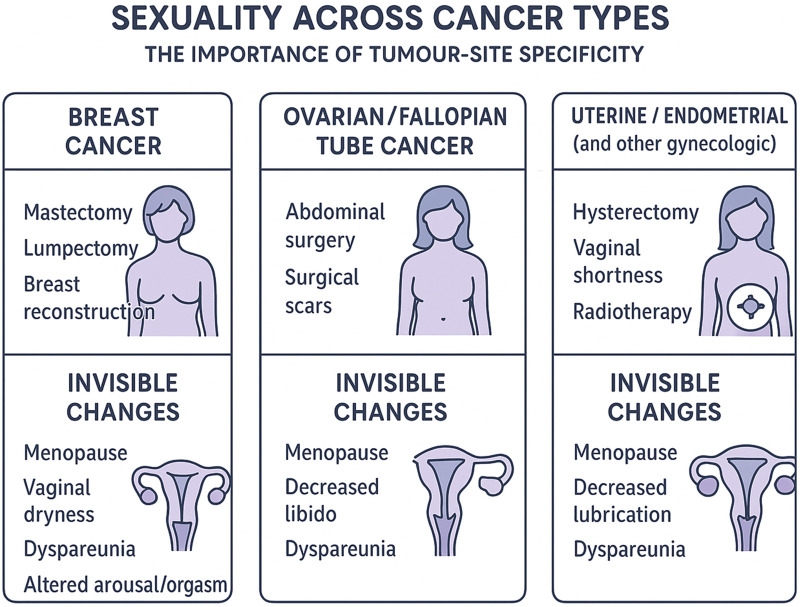
Multidimensional framework of sexual health in cancer survivors. This schematic highlights the interconnected domains that shape sexual wellbeing after cancer: physical changes (e.g., pain, neurovascular injury, genitourinary symptoms), hormonal alterations, psychosocial factors (body image, mood, identity), relational dynamics, and fertility-related concerns. Male-specific, female-specific, and shared factors are visually distinguished. Surrounding these core domains are key intervention areas—including medical and pharmacologic treatments, physical rehabilitation, psychosexual and couples therapy, and structured communication pathways—that together support comprehensive, patient-centered sexual healthcare across the cancer trajectory.

Sexuality in cancer survivorship is far more than a comfort measure—it represents an essential dimension of identity, intimacy, and long-term quality of life. Figure 4 summarizes the multidimensional nature of sexual health after cancer, illustrating how physical, hormonal, psychosocial, relational, and identity/fertility domains intersect and how targeted interventions operate across these layers. Cancer treatments disrupt sexual wellbeing through physical, hormonal, relational, and identity-related pathways. Younger patients are particularly affected, as fertility loss intersects with sexual identity and future planning, highlighting the need for fertility preservation discussions integrated with sexual counselling (76). Many survivors experience invisible changes—internal anatomical or neurovascular alterations, reduced sensation, diminished arousal or orgasm, and shifts in sexual self-concept—that may remain unrecognized. Sexuality after cancer is also influenced by cultural, gender, and orientation contexts, with sexual and gender minority survivors facing distinct vulnerabilities and lower satisfaction with care when identity is not addressed (77). Sexual health must therefore be embedded across all phases of oncology care, with tumor-specific screening and interventions, multidisciplinary teams, communication models, partner support, and survivorship planning. Sexual medicine clinicians are uniquely positioned to bridge oncology and survivorship care by addressing the physical, emotional, and relational dimensions of sexuality after cancer.”
